# Management of primary choroidal lymphoma presenting as extensive serous retinal detachment and salmon patch of bulbar conjunctiva: a case report

**DOI:** 10.1186/s13256-022-03373-z

**Published:** 2022-04-24

**Authors:** Zahra Mahdizad, Elias Khalili Pour, Mohammadreza Mehrabi Bahar, Amirreza Esfandiari, Babak Masoomian, Hamid Riazi-Esfahani, Ahmad Mirshahi, Fariba Ghassemi

**Affiliations:** 1 Retina Service, Farabi Eye Hospital, Tehran University of Medical Sciences, Tehran, Iran; 2 Ocular Oncology Department, Farabi Eye Hospital, Tehran University of Medical Sciences, Tehran, Iran

**Keywords:** Lymphoma, Marginal zone B-cell lymphoma radiotherapy, Reactive lymphoid hyperplasia, Uveal neoplasms

## Abstract

**Background:**

We describe the outcome of ultra-low-dose radiotherapy plus intravitreal methotrexate and rituximab injections for a patient with primary choroidal lymphoma who presented with nodular conjunctival salmon patches and extensive serous retinal detachment.

**Case presentation:**

A 34-year-old Iranian man presented with a nodular patch of bulbar conjunctiva in the right eye, and 1+ vitritis. A nearly complete shallow serous retinal detachment, retinal folds, and multifocal yellow choroidal infiltrates were seen during a fundus examination of the right eye. Enhanced depth imaging optical coherence tomography revealed macular retinal folds and an uneven, undulating, “seasick” appearance of the choroidal surface with choriocapillaris compression, intraretinal and subretinal fluid, and clusters of optically dense material at the outer retinal level. An incisional biopsy of the conjunctival lesion confirmed the diagnosis of primary choroidal lymphoma with epibulbar involvement. The patient was treated with ultra-low-dose “boom-boom” radiation (4 Gy delivered in two fractions over two consecutive days) as well as intravitreal methotrexate and rituximab injections. After a year, the lesions had completely disappeared, with no adverse effects or recurrence.

**Conclusion:**

Ultra-low-dose (boom-boom) radiotherapy combined with intravitreal chemotherapy and/or immunotherapy may be an effective treatment for primary choroidal lymphoma with anterior epibulbar extension and diffuse subretinal fluid with favorable response and minimal side effects.

## Background

Primary choroidal lymphoma (PCL) is an indolent, low-grade tumor, previously known as reactive lymphoid hyperplasia (RLH) or uveal pseudotumor. It is typically classified as extranodal marginal zone B-cell lymphoma when there is no evidence of systemic illness at the time of diagnosis [1]. Owing to its rarity and the fact that many ophthalmologists are unfamiliar with it, primary choroidal lymphoma is commonly misdiagnosed as a variety of other disorders [2].

Observation, radiation, chemotherapy, and immunotherapy (with rituximab) are all options for treating choroidal lymphoma [3]. Given choroidal lymphoma’s rarity, the appropriate radiation dose is debatable. Low-grade lymphoma is often treated with conventionally fractionated radiation doses ranging from 25 to 36 Gy. Recently, there has been a trend to treat PCL with extremely low radiation doses, as low as two consecutive 2 Gy fractions (referred to as “boom-boom radiotherapy”) [3–5].

We present what we believe to be a rare case of primary choroidal lymphoma that presented with nodular salmon patches in the bulbar conjunctiva of the right eye and extensive serous retinal detachment, which responded well to ultra-low-dose radiation along with intravitreal chemotherapy and immunotherapy.

## Case presentation

**Fig. 1:**
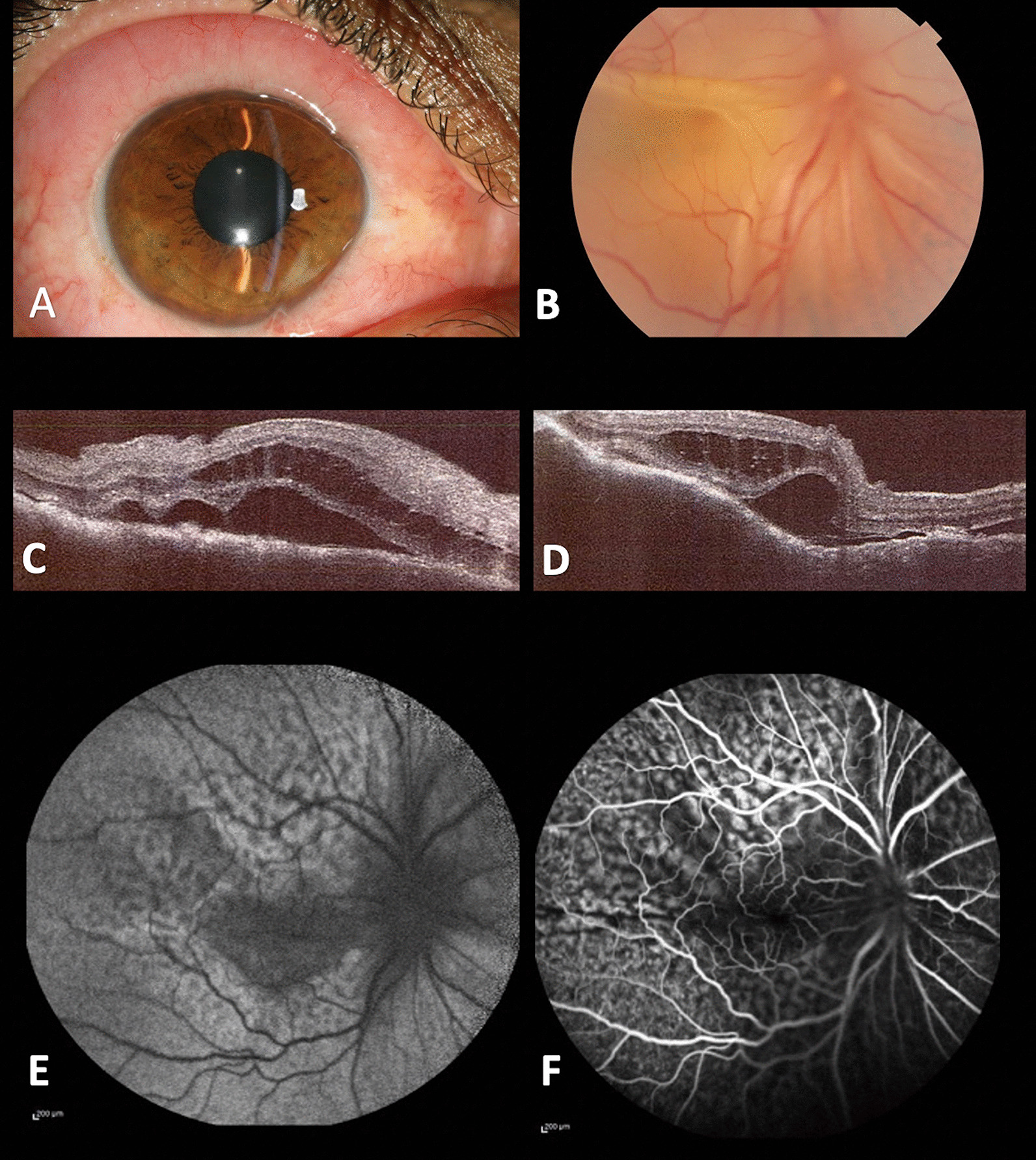
**A** Chemosis and a salmon-pink nodular patch involving the bulbar conjunctiva in the right eye were discovered during a slit-lamp examination, as well as 1+ vitritis. **B** The fundus examination of the right eye revealed shallow serous retinal detachment, chorioretinal fold, and widespread mid-peripheral retinal pigment epithelium (RPE) changes. **C**, **D** Corresponding horizontal and vertical enhanced-depth-imaging optical coherence tomography slabs revealed macular retinal folds and an uneven, undulating, “seasick” appearance of the choroidal surface with choriocapillaris compression, intraretinal and subretinal fluid, and many clusters of optically dense material at the RPE level. **E**, **F** Blue autofluorescence and late-phase fluorescein angiography imaging revealed a nonspecific "leopard-spot" pattern

A 34-year-old Iranian man presented to the clinic with redness and impaired vision in the right eye for many months, despite topical corticosteroid treatment. His prior medical history was unremarkable. His best-corrected visual acuity (BCVA) in the right eye was 4/10 (+5.00 sphere) and 10/10 (−0.50 sphere) in the left eye at the time of presentation. The examination of the left eye was normal. During a slit-lamp examination, the right eye revealed chemosis as well as a nodular salmon patch in the bulbar conjunctiva and 1+ vitritis. The fundus examination of the right eye revealed near-total shallow serous retinal detachment, multifocal yellow choroidal infiltrates, and widespread mid-peripheral retinal pigment epithelium (RPE) changes (Fig. 1A, B).

Enhanced-depth-imaging optical coherence tomography (EDI-OCT) showed macular retinal folds and a “seasick” appearance on the choroidal surface with compression of the choriocapillaris. OCT images also revealed intraretinal and subretinal fluid, as well as clumps of optically dense material at the level of the RPE (Fig. 1C, D).

Fluorescein angiography (FA) disclosed diffuse patches of choroidal hyperfluorescence. In the posterior pole, blue autofluorescence (BAF) and FA imaging exhibited a nonspecific “leopard-spot” appearance (Fig. 1E, F).

Incisional biopsy of the conjunctival lesion was performed; histological and immunohistochemistry studies revealed a dense infiltrate of small lymphocytes with positive staining for CD20 and CD79, with coexpression of BCL2. The diagnosis was atypical monoclonal lymphoid infiltration suggestive of low-grade extranodal marginal zone B-cell lymphoma.

Magnetic resonance imaging (MRI) of the orbit was negative for extrascleral extension; his systemic workup for extraocular involvement including laboratory data, chest and abdomen computed tomography (CT) scans, and bone marrow aspiration results were all negative.

Ultra-low-dose radiation, termed “boom-boom radiotherapy” (4 Gy delivered in two fractions over two consecutive days) was used in conjunction with intravitreal injections of methotrexate and rituximab. To reduce radiation scatter to periocular tissue, orbital rim bones, and midfacial soft tissues, the beam was delivered through a bolus shell of 42-mm water-equivalent thickness placed 3 cm from the patient.

Intravitreal methotrexate injections were started at a dosage of 400 µg in 0.1 ml per week for 6 weeks, then monthly for 6 months. Rituximab was administered intravitreally once a month for 3 months at a dosage of 1 mg in 0.1 ml. The intravitreal injections were started just 1 week after the last radiotherapy session.

**Fig. 2:**
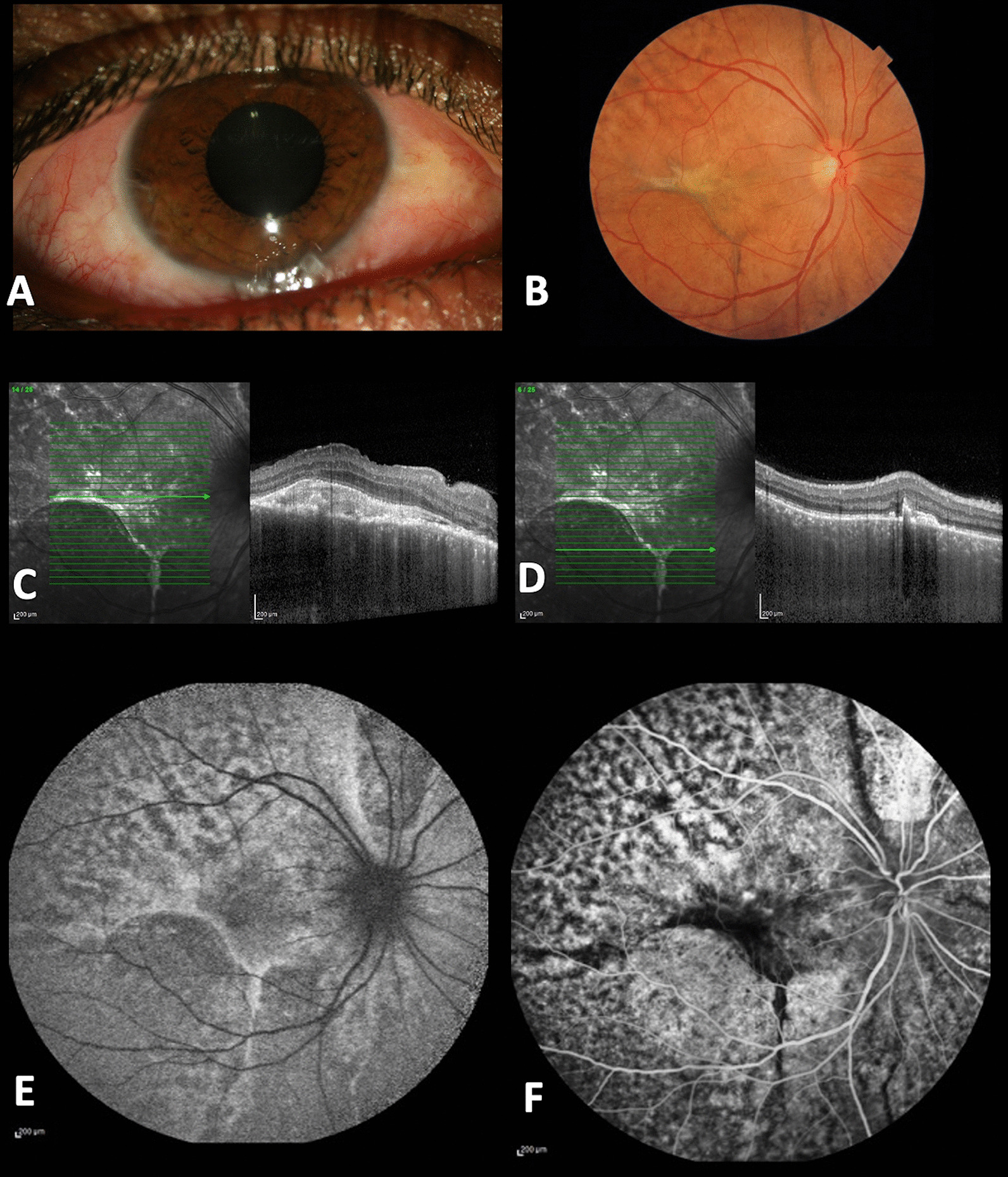
**A** Two months following therapy, a slit-lamp examination of the right eye revealed resolved chemosis and a salmon-pink nodular patch of bulbar conjunctiva. **B** Fundus examination revealed subretinal fibrosis and extensive RPE changes at the site of previous prolonged retinal detachment. **C**, **D** Corresponding foveal and perifoveal horizontal optical coherence tomography B-scans show resolution of lymphoid infiltration, flattening of the macular fold, and clearance of subretinal and intraretinal fluids, as well as subretinal hyperreflective deposits that matched to areas of subretinal fibrosis. **E**, **F** Blue autofluorescence and late-phase FA imaging revealed “leopard spots,” as well as the addition of hypofluorescent lines in FA correlating with subretinal fibrosis

Serous retinal detachment was resolved 2 months following ultra-low-dose radiation, and subretinal fibrosis and widespread RPE alterations were observed at the location of the detachment. On EDI-OCT, the lymphoid infiltration, flattening of the macular fold, and resolution of subretinal and intraretinal fluid were all observed. Hyperreflective deposits were observed under the retina that correlated to fibrosis. The RPE–choroidal interface changed to a “calm sea” appearance. (Fig. 2A–F)

At 1-year follow-up, the results remained consistent, the subconjunctival lesions were regressed, and the BCVA improved to 6/10. During this time, there was no recurrence or radiation-associated complications. The patient was scheduled for regular follow-up visits.

## Discussion and conclusions

This is a rare case of primary choroidal lymphoma characterized by choroidal infiltration, serous retinal detachment, and retinal folds with adjacent extraocular extension. However, there was 1+ cellular infiltration in the vitreous cavity, which is unusual in individuals with primary uveal lymphoma [2]. We diagnosed choroidal lymphoma on the basis of the presence of ocular adnexal (conjunctiva) involvement and its pathologic features.

The most frequent presenting symptom of primary choroidal lymphoma is impaired vision; however, around 9–20% of patients are asymptomatic [2, 6]. The presence of multifocal yellow-white patches at the level of choroid is possibly the most useful fundoscopic finding for establishing the clinical diagnosis of PCL. Aronow *et al.* reported choroidal yellow-white infiltrates as the main fundus finding in all patients with choroidal lymphoma in their study (22 patients) [6]. Multifocal yellow-white subretinal infiltrates are also common in primary vitreoretinal lymphoma eyes; however, these infiltrates are often seen in the subretinal space or between the RPE and the Bruch’s membrane rather than in the choroid [7–9]. Furthermore, our patient had outer retinal involvement as conglomerates of highly reflective foci.

The EDI-OCT may therefore prove valuable in illustrating lymphoma-related choroidal infiltrations in patients with intraocular lymphomas, in addition to evaluating their severity before and after treatment [10]. Thin choroidal infiltrations (1–2 mm) have a smooth or calm look, but tumors with a thickness of 2–3 mm have a rippled appearance, and lesions with a thickness of more than 4 mm have an undulating or seasick appearance [11].

Indicative of PCL, the patient had a diffuse widespread shallow serous retinal detachment in all quadrants. Even though PCL is widespread, serous retinal detachment is rare and, if present, is shallow and limited [2]. Mashayekhi *et al.* found in a case series that half of the 49 patients with PCL did not have retinal detachment, and only 19% had serous retinal detachment involving two or more quadrants, including 3 (6%) with subretinal fluid in all four quadrants [2]. In another study, Arnow *et al.* found 3 eyes (10.3 %) out of 34 with subretinal fluid [6]. However, nonlymphomatous choroidal metastases usually cause more extensive serous retinal detachment.

The presence of cellular response in the vitreous cavity is uncommon in patients with PCL (2%) [2]. In contrast, almost 30% of eyes with secondary choroidal lymphoma (6 out of 24) showed signs of vitritis at baseline. As a result, vitreous cellular infiltration has been observed to be indicative of secondary choroidal lymphoma, and systemic workups in these individuals should be performed at regular intervals [2]. The vitreous reaction in our patient raises the possibility of secondary involvement of choroids from primary adnexal lymphoma.

According to some studies, the sessile salmon patch formed by uveal lymphoid infiltrations outside the sclera is seen in individuals with choroidal lymphoma (up to 40%) [2, 6, 11]. The patches under the conjunctiva not only facilitate early diagnosis, but they are also useful for biopsies. [12–15]

Mashayekhi *et al.* found that more than half of patients exhibited posterior epibulbar lymphoid cell expansion [6]. As a result, a B-scan may be required to locate a suitable biopsy site, especially in the lack of anterior epibulbar extension [2].

Because of the rarity of PCL, there is no recognized standard treatment approach; nevertheless, most experts prefer external beam radiation (EBRT) for localized uveal lymphoma and chemotherapy and immunotherapy with rituximab in cases of multifocal or systemic disease [6, 16]. EBRT with standard dosages (25–36 Gy) was the most commonly used treatment for choroidal lymphoma without concurrent systemic lymphoma, yielding good response rates and excellent local control. However, radiation toxicity can affect radiosensitive ocular structures, including the cornea, lens, retina, and optic nerve, particularly in people with predisposing conditions such as diabetes mellitus or other retinal vascular disorders, or those who are receiving concurrent systemic chemotherapy [17]. Konig *et al.* compared high-frequency radiotherapy and conventional radiotherapy for treating orbital lymphoma and observed that ultra-low-dose therapy had less radiation-related effects than conventional radiotherapy, even though both treatments were effective [18]. Later, it was demonstrated that ultra-low radiation doses were beneficial in the treatment of individuals with localized primary choroidal lymphoma [3, 19]. We used low-intensity radiation (boom-boom radiotherapy) and intravitreal injections of methotrexate and rituximab in the current case because of the presence of cellular infiltration in the vitreous cavity, which is an unusual symptom of PCL [2]. Despite a significant response to these therapies just 2 months after treatment, large studies are needed to determine whether intravitreal injections are useful additions to EBRT for patients with PCL and vitritis.

In conclusion, we reported the case of a patient with biopsy-proven primary choroidal lymphoma who had severe serous retinal detachment in all quadrants as well as vitritis and was treated with ultra-low-dose “boom-boom” radiation, intravitreal methotrexate, and rituximab injections. After 1 year, the tumor had completely resolved with no adverse effects or recurrence.

## Data Availability

All data generated during this study are included in this published article.

## Abbreviations

PCL: Primary choroidal lymphoma
RLH: Lymphoid hyperplasia
BCVA: Best-corrected visual acuity
RPE: Retinal pigment epithelium
EDI-OCT: Enhanced-depth-imaging optical coherence tomography
FA: Fluorescein angiography
BAF: Blue autofluorescence
MRI: Magnetic resonance imaging
CT: Computed tomography
EBRT: External beam radiation
